# A homogeneous bioluminescent immunoassay for parallel characterization of binding between a panel of antibodies and a family of Fcγ receptors

**DOI:** 10.1038/s41598-022-15887-z

**Published:** 2022-07-16

**Authors:** Nidhi Nath, Becky Godat, Rod Flemming, Marjeta Urh

**Affiliations:** 1 Promega Corporation, R&D Department, 2800 Woods Hollow Road, Madison, WI 53711 USA; 2 Present Address: Bio-Techne, R&D Department, 614 McKinley Place NE, Minneapolis, MN 55413 USA

**Keywords:** Biological techniques, Immunology

## Abstract

Fc engineering efforts are increasingly being employed to modulate interaction of antibodies with variety of Fc receptors in an effort to improve the efficacy and safety of the therapeutic antibodies. Among the various Fc receptors, Fc gamma receptors (FcγRs) present on variety of immune cells are especially relevant since they can activate multiple effector functions including antibody dependent cellular cytotoxicity (ADCC) and antibody dependent cellular phagocytosis (ADCP). Depending on the desired mechanism of action (MOA) of the antibody, interactions between Fc domain of the antibody and FcγR (denoted as Fc/FcγR) may need to be enhanced or abolished. Therefore, during the antibody discovery process, biochemical methods are routinely used to measure the affinities of Fc/FcγR interactions. To enable such screening, we developed a plate based, simple to use, homogeneous immunoassays for six FcγRs by leveraging a luminescent protein complementation technology (NanoBiT). An added advantage of the NanoBiT immunoassays is their solution-based format, which minimizes well known surface related artifacts associated with traditional biosensor platforms (e.g., surface plasmon resonance and biolayer interferometry). With NanoBiT FcγRs assays, we demonstrate that assays are specific, report IgG subclass specific affinities and detect modulation in Fc/FcγR interactions in response to the changes in the Fc domain. We subsequently screen a panel of therapeutic antibodies including seven monoclonal antibodies (mAbs) and four polyclonal intravenous immunoglobulin (IVIg) products and highlight the advantages of parallel screening method for developing new antibody therapies.

## Introduction

Antibody driven therapies have made significant impact on the treatment of multiple diseases including cancer, autoimmune, metabolic, viral, and infectious diseases. Therapeutic monoclonal antibodies have become a mainstay for treatment of cancer and other diseases because they bind their target with high specificity and affinity recruiting various effector cells through Fc receptor interaction resulting in target elimination^1^. Another modality in which antibody therapy has proven beneficial is the intravenous IgG (IVIg) therapy. IVIg contains polyclonal immunoglobulin G (IgG) from thousands of donors, and it is administered at low dose (200–400 mg/kg) as antibody replacement therapy to individuals who can’t make antibodies due to B-cell related malignancies. IVIg at high dose (1–2 g/kg) is used in the treatment of auto-immune or auto-inflammatory diseases and acts by modulating immune function through engagement with a variety of Fc receptors^2^. Even though the exact mechanism of action of IVIg remains to be understood, the effectiveness of this therapy is evident by its extensive use against a broad range of indications with demand growing 6–8% annually^3^. Finally, the impact of antibodies in infectious disease management has been made abundantly clear by the success of safe and effective vaccines against COVID-19 in containing the spread of SARS-CoV-2 infection. COVID-19 vaccines induce a robust antibody response including neutralization antibodies blocking the entry of virus into the cells but also has a secondary impact of activating effector functions through interaction with a variety of Fc gamma receptors (FcγRs) on immune cells including natural killer cells and macrophages^4–6^.

IgG molecule is composed of two identical fragment antigen binding domains F(ab)_2_ and one fragment crystallizable (Fc) domain. F(ab)_2_ containing variable light and heavy chains are responsible for antigen detection. The Fc domain on the other hand determines the fate of antigen–antibody complex by binding to the FcγRs on various immune cells and triggering effector functions such as antibody dependent cellular cytotoxicity (ADCC) and antibody dependent cellular phagocytosis (ADCP). Recruitment of effector cells depends on several factors including the IgG subclass (e.g. IgG1-4), Fc domain glycosylation, and Fc/FcγR binding affinity. Fc/FcγR denotes the interaction of IgG as well as Fc fusion proteins with FcγRs. Five different Fcγ receptor subclasses are known to be present on human effector cells FcγRI (CD64), FcγRIIa, (CD32a), FcγRIIb (CD32b), FcγRIIIa (CD16a), and FcγRIIIb (CD16b)^7,8^. Different polymorphic variants of some of these receptors exists, for example FcγRIIIa is present as V158 and F158 with the former exhibiting higher affinity for IgG compared to the latter. Similarly, there are two known variants of FcγRIIa, H131 and R131. Several excellent reviews cover the relevance of these receptors for therapeutic antibodies and therefore are not discussed here^8,9^. In addition to FcγRs expressed on the cell membrane of immune cells, there is an additional Fc receptor called the neonatal Fc receptor (FcRn), which is present in endosomes and binds the Fc domain at acidic pH and determines the half-life of IgGs in the bloodstream^10^. It is worth noting that FcRn and FcγR bind on mutually exclusive binding site of the Fc domain.

It is routine to make rational modifications in the Fc domain of antibodies to modulate interactions with FcRn and FcγRs and achieve certain therapeutic goals. For example, Fc/FcRn interactions can be modulated to improve the half-life of the antibodies whereas Fc/FcγRIIIa interaction can be modified to enhance or in some cases abolish the ADCC function. Considering the importance of Fc/FcγR interactions, the methods to measure these interactions are fairly limited with the predominant method being the use of biosensors such as Surface Plasmon Resonance (SPR) and Biolayer interferometry (BLI)^11^. Biosensor platforms require immobilization of one protein on the chip surface followed by incubation with the binding partner and monitoring of association and dissociation rate from which affinity constants can be derived. Though extremely powerful and widely used, these methods do have limitations as affinity measurements are influenced by the assay format, sensor chip characteristic, and immobilization method^12^. Such limitations have been extensively investigated for measurement of Fc/FcRn interactions^13^, and studies have proposed guidelines to minimize artifacts and obtain accurate results, but the time and resources required to pursue all these options are significant and such guidelines may not be generalizable. The later aspect is evident in the different recommendations made by these studies, which were specific to the model antibody tested in the study. Similar detailed investigation on surface impact on Fc/FcγR is lacking and may be one of the reason for widely divergent affinity values reported in the literature^11^. In addition to biosensor platforms, two bead-based assays have also been used; first, the Luminex microsphere assays developed for high-throughput characterization of Fc/FcγR interactions^14,15^ and second, the AlphaLISA proximity assay using beads for measurement of antibody binding with FcγR^16,17^. Assays performed using beads allow for high-throughput assays with small sample volumes, but still require surface immobilization which has disadvantages.

**Figure 1:**
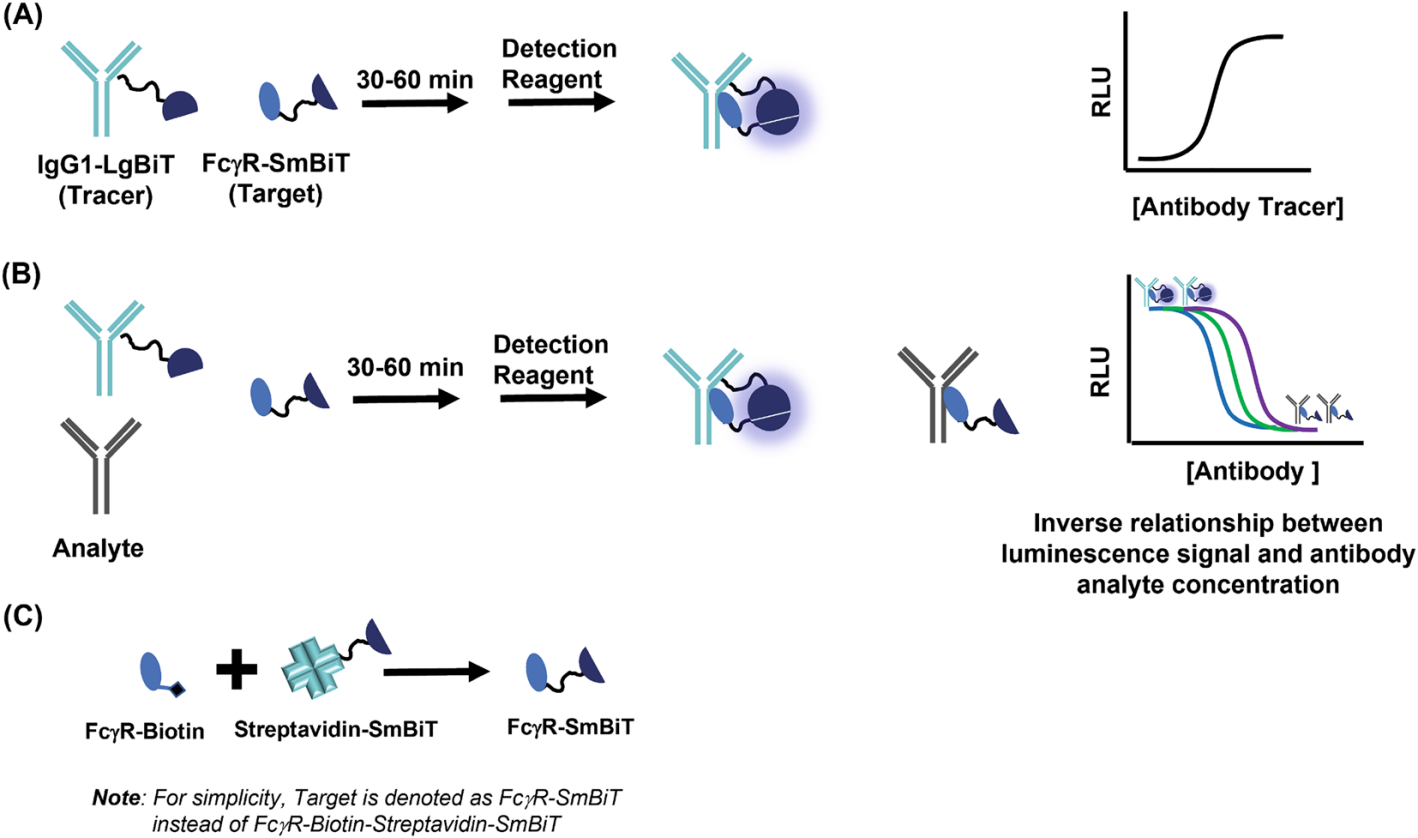
Schematic showing development of NanoBiT FcγR immunoassay. (**A**) Tracer-Target binding assay is performed to determine their optimum amount and provide IC_50_ values that are equivalent to dissociation constant (K_D_). (**B**) Workflow of the NanoBiT FcγR competition assay to calculate IC_50_ values of the analyte. (**C**) Modular approach to the formation of various FcγR-SmBiT tracer by combining biotinylated FcγR with Streptavidin-SmBiT.

To alleviate the surface and immobilization related artifact and offer an orthogonal assay, we recently reported a bioluminescent homogeneous immunoassay (Lumit) based on NanoBiT protein complementation technology for measurement of Fc/FcRn interactions^13^. Briefly, NanoBiT luminescent enzyme is composed of a 11-aa peptide called Small BiT (SmBiT) and a 18-kDa polypeptide called Large BiT (LgBiT) that have a very weak affinity (K_D_ > 100 µM) for each other but when forced into close-proximity they form a bright bioluminescent enzyme. Apart from being solution-based, these assays eliminate any washing steps and are therefore easy and rapid. Immunoassays using NanoBiT technology have been used in multiple application such as the detection of protein interactions, SARS-CoV-2 antibody detection and neutralization assays, as well as in the development of Fc/FcRn interactions among others^18–21^. In this work, we leveraged the approach used in measuring Fc/FcRn interactions and describe assay for the measurement of Fc interaction with family of six Fcγ receptors namely—FcγRI, FcγRIIa (H131), FcγRIIa (R131), FcγRIIb, FcγRIIIa (V158), and FcγRIIIa (F158) (Fig. 1A). We first validated that NanoBiT FcγR assays are specific, can bind antibodies in a subclass-specific manner and can respond to changes in the IgG Fc domain. Subsequently a collection of therapeutic mAbs, containing seven antibodies of various IgG subclasses and one Fc fusion protein, against a variety of antigens were screened against all six FcγRs. Our analysis revealed affinities of IgG molecules that matches their subclass; however, Fc fusion protein did show results different from the traditional IgG molecules. A subset of mAbs were also tested on a BLI platform and, in general, there was good correlation between BLI and NanoBiT platform except for the Fc fusion protein. In addition, we tested a panel of four IVIg drugs, which are polyclonal antibodies and will mimic the *in-vivo* effect on receptors. Not surprisingly, all four IVIg drugs had similar binding to six FcγRs but affinities were different than what was observed with monoclonal antibodies. Finally, we combined all the data and highlight the advantages of the NanoBiT immunoassay platform in accelerating the discovery of new therapeutic modalities of antibody drugs by enabling parallel measurement of binding affinities of a large panel of antibodies against a family of FcγRs.

## Materials and methods

### Materials

Recombinant FcγRI, FcγRIIa (H131), FcγRIIa (R131), FcγRIIb, FcγRIIIa (V158), and FcγRIIIa (F158) with C-terminus Biotin were from ACROBiosystems (Newark, DE). Human IgG1 used for labeling with LgBiT was produced by GlycoScientific (Atlanta) and had a sequence similar to Adalimumab (https://go.drugbank.com/drugs/DB00051). Streptavidin was from Sigma. Antibodies tested using NanoBiT FcγR assays are listed in the Table 1. Labeling was performed using Lumit Immunoassay labeling kit (Promega). Buffers were prepared in-house, and Superblock was purchased from ScyTek. 96-well U-bottom white polypropylene plates (Eppendorf) were used for all the experiments and luminescence was read on Glomax Discover (Promega).

**Table 1:** List of antibodies used in this work.

Antibody#	Antibody description	Company	Catalog#
1	Human IgG1	Athens Research & Technology	16-16-090707-1
2	Human IgG2	Athens Research & Technology	16-16-090707-2
3	Human IgG3	Athens Research & Technology	16-16-090707-3
4	Human IgG4	Athens Research & Technology	16-16-090707-4
5	Human IgG	Rockland Immunochemical	009-0102
6	Human IgG Fc Fragment	Jackson ImmunoResearch	009-000-008
7	Human IgG, Fab Fragment	Rockland Immunochemical	009-0104
8	Human IgG, F(ab)_2_ Fragment	Rockland Immunochemical	009-0105
9	Human Serum Albumin (HSA)	Sigma	A8763
10	Infliximab	Janssen	NDC 67894-030-01
11	Etanercept	Immunex Corporation	NDC58406-455-01
12	Adalimumab	Evidentic GmbH	8000152
13	Rituximab	Evidentic GmbH	8000100
14	Cetuximab	Lilly	NDC-66733-948-23
15	NIST	Sigma	8671
16	Panitumumab	Amgen	NDC 55513-954-01
17	Pembrolizumab	Evidentic GmbH	8000933
18	Flebogamma	Griflos	NDC 61953-0005-2
19	Gamunex	Griflos	NDC 13533-800-12
20	Octagam	Octapharma	NDC 68982-840-03
21	Gammagard	Shire	NDC 0944-2700-02
22	Anti-hCD20-hIgG1	Invivogen	hCD20-mab1
23	Anti-hCD20-hIgG1fut	Invivogen	hCD20-mab13
24	Anti-hCD20-hIgG1NQ	Invivogen	hCD20-mab12

### Labeling of IgG and Streptavidin with LgBiT and SmBiT

The labeling of IgG and Streptavidin with LgBiT and SmBiT respectively was done according to vendor suggested protocol and has been described in detail before^13^. Briefly, solution of Human IgG1 and Streptavidin at 1.0 mg/mL were pH adjusted (pH 8.3) with 1/10th volume of 1.0 M bicarbonate buffer (pH 8.3–8.5) and reacted with 20-fold molar excess of amine reactive HaloTag Ligand (HaloTag® Succinimidyl Ester (O4) Ligand, Promega) for 1 h. Unreacted HaloTag ligand was removed with Zeba desalting column (Thermofisher Scientific). IgG1 and Streptavidin activated with HaloTag ligand were incubated overnight with four molar excesses of HaloTag-LgBiT or HaloTag-SmBiT fusion protein, respectively. Any unreacted HaloTag-LgBiT or HaloTag-SmBiT were removed by incubating the samples with HaloLink beads (Promega). Non-reducing SDS-PAGE gel was used to confirm the labeling of Streptavidin and IgG.

### NanoBiT FcγR binding immunoassay

NanoBiT FcγR binding immunoassays involves four steps. In the first step, 25 (8) µL of tracer solution (IgG-LgBiT) was mixed with 25 (8) µL of the sample (antibody or Fc fusion protein) in a white 96 well plate (Corning). Numbers in the parentheses are the volumes used in scaled down assays. In the second step, FcγR-Biotin and Streptavidin-SmBiT were mixed at 1:1 molar ratio to form the target, FcγR-Biotin-Streptavidin-SmBiT (FcγR-SmBiT). In the third step, 50 (16) µL of target was added to each well containing sample and the tracer and reaction was incubated for 30–60 min, to allow binding of target to either the analyte antibody in the sample or to the tracer. In the final step, Furimazine substrate (Promega) was diluted 1:50 fold and 25 (8) µL was added to plate. The signal was allowed to stabilize for 3 min, after which the bioluminescence signal (RLU) was measured in a Glomax Discover Luminometer. All the dilutions were made in phosphate buffer saline (PBS) containing 10% superblock. The only exception to this setup was the assay for FcγRIIb. Due to the very weak affinity (in µM) of the receptor for IgG, FcγRIIb-Biotin tag and Streptavidin-SmBiT were mixed at 4:1 molar ratio to achieve sufficient signal/background ratio and have a robust assay.

Normalized RLU data was generated by assigning 100% to the maximum bioluminescent signal obtained in absence of the sample and then calculating percentage drop in signal in the presence of the sample. Inhibition curves were generated by plotting normalized RLU as a function of the sample concentration (in nM) and fitted to a four parametric equation with 1/y^2^ weightage using GraphPad Prism. IC_50_ (nM) values which are equivalent to apparent affinity values (K_D_) were used when comparing data generated using biosensor and reported in the literature. The Student’s t test was used to compare IC_50_ values. In all cases P-values < 0.05 were considered to indicate statistical significance.

### Biolayer interferometry (BLI) for Fc/FcγR affinity measurement

Binding of antibodies to biotinylated FcγR was measured using BLI on Octet Red system (Pall Bio). Protocols for the assay were kindly provided by Acro Biosystems. FcγR-AviTag proteins were loaded on to the Streptavidin coated biosensor to a final surface density of ~ 0.5–1.0 nm. For FcγRI, which is a high affinity receptor, two-fold dilution of antibody or Fc fusion protein from to 75 nM-1.17 nM along with a blank were prepared with PBST (PBS containing 0.02% Tween20) at pH 7.2. For rest of the receptors a two-fold dilution from 5000 to 78.125 nM and a blank were used. Samples were incubated with FcγR-AviTag-coated biosensor for 60 s to measure the association rate followed by 60 s wash in PBST to measure dissociation. All data were plotted using instrument software and steady state data were plotted on GraphPad Prism and fitted to a one-site binding curve to calculate the equilibrium affinity constant K_D_.

## Results

### Optimization of NanoBiT FcγR binding immunoassay

Our goal was to develop a solution based, simple to use, reproducible, and high-throughput assay to enable screening of a panel of antibodies against multiple FcγRs. We therefore decided to use the NanoBiT based bioluminescent competition immunoassay format recently developed by our team for monitoring antibody interaction with FcRn. The assay format involves interaction between a FcγR-SmBiT (target) and IgG1-LgBiT (tracer) resulting in a luminescence signal (Fig. 1A). The presence of a sample containing Fc domain (antibodies or Fc fusion protein) results in competitive inhibition of the tracer binding to the target which leads to a decrease in luminescence signal (Fig. 1B). Assay format is also modular, means that simply by switching FcγR-Biotin (Fig. 1C), while keeping rest of the reagents same, allowed us to create six different assays as described below. A point worth noting is that IgG1-LgBiT and Streptavidin-SmBiT used in the assay were prepared by chemical labeling of IgG1 and Streptavidin with LgBiT and SmBiT using amine chemistry and we estimated a labeling efficiency of 3–4 BiTs per molecule of IgG and Streptavidin. The amine chemistry of labeling proteins and antibodies with fluorophore, biotin, and enzymes is widely used and is also the method of choice in making of FDA approved antibody drug conjugates^22^. A disadvantage of chemical labeling method is its stochastic nature in which the number and position of label cannot be controlled, and the nature and size of the label can impact the protein functionality. Therefore, we made a robust protocol and generated multiple batches of IgG-LgBiT as well as Streptavidin-SmBiT described in our previous work^13^ and obtained very reproducible results.

**Figure 2:**
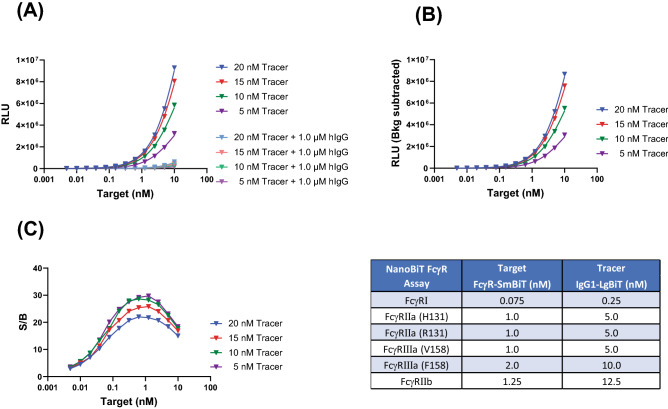
Representative data for the tracer-target titration shown for FcγRIIIa (V158). (**A**) Concentration dependent increase in luminescent signal as function of FcγRIIIa (V158)-SmBiT target and IgG-LgBiT tracer. Presence of excess human IgG competes with the tracer resulting in decrease in luminescent signal. Luminescent signal in the presence of excess human IgG is considered background and arises from random interaction between SmBiT and LgBiT. Data was generated in a 96-well plate in single replicate. (**B**) Background subtracted luminescence signal represent specific interaction between target and the tracer. (**C**) Signal/background (S/B) ratio is calculated at each tracer target concentration and a specific concentration of tracer and target is selected (shown in the Table) for subsequent assays that satisfies the Cheng-Prusoff equation and offers high luminescence signal so that assay can be performed on any luminometer.

Optimization of the assay required meeting several key criteria: (a) the requirement of the Cheng-Prusoff equation^23^ of target concentration < tracer concentration < apparent dissociation constant (K_D_) (under these conditions, IC_50_ values obtained from the competitive assay will correspond to the apparent affinity values); (b) wide assay window; and (c) high luminescence signal so that assay can be performed on variety of Luminometers. We chose NanoBiT Fc/FcγRIIIa immunoassay to demonstrate that using this approach we can achieve the requirements listed above. Published data using traditional biosensor platforms have reported affinity values (K_D_) of IgG/FcγIIIa (V158) in the range of 7.1 nM-2.0 µM^11^. Considering this wide range of reported affinity values, it was hard to decide on a starting point for tracer and target titration needed to meet the requirements of Cheng-Prusoff equation. Thus, based on our experience, we decided on using a two-fold dilution series of target from 10 nM-0.005 nM and tracer concentration of 20 nM, 15 nM, 10 nM, and 5 nM. A checkerboard experiment involving titration of target and tracer was performed as shown in Fig. 1A, and a concentration dependent increase in luminescent signal was observed (Fig. 2A) To ensure that tracer-target binding is specific, another checkerboard experiment was performed in the presence of excess human polyclonal IgG (1.0 µM). If interaction between the target and tracer is specific, then excess of Fc containing IgG will compete with the Fc of the tracer binding to the target, resulting in drop in bioluminescent signal as observed (Fig. 2A). Low level of bioluminescent signal in presence of excess IgG is background arising from random interaction between LgBiT and SmBiT. Background subtracted luminescence signal shown in Fig. 2B is the bioluminescence value arising from specific tracer-target binding. A ratio of signal/background (S/B) was also plotted (Fig. 2C) and is a measure of assay window. The Tracer concentration of 5.0 nM and target concentration of 1.0 nM meets the requirements of Cheng-Prusoff equation and gave us a S/B of around 29-fold with high luminescence signal and was selected for competition assays.

Similar data were generated for binding of tracer with other FcγR-SmBiT targets (data not shown). By applying the criteria mentioned above to the data set, concentrations of tracer and target shown in the Table (Fig. 2) were used in the competition assay to determine the binding affinities of the panel of Fc containing molecules. It is important to point out two exceptions to the requirement pointed in the previous section. First exception was the assay for FcγRI, where due to the high affinity (0.12–52 nM), it was not possible to use the tracer and target concentration lower than the K_D_ values since it led to very low luminescence signal. For FcγRI, tracer and target concentrations of 0.25 and 0.075 nM respectively was selected which in many cases exceeded the affinity values obtained using NanoBiT as shown in later section. Second exception is the assay for FcγRIIb where due to very weak micromolar affinity^11^ with IgG, sufficient S/B ratio was not obtained and assay was not reproducible. Increasing the ratio of FcγRIIb-Biotin tag and Streptavidin-SmBiT to 4:1 molar ratio improved the S/B possibly due to the avidity effect. This approach of making tetrameric complex has also been used in bead assays to capture weak binding of IgG to FcγRII and FcγRIII^15^. As a result, IC_50_ values for FcγRI and FcγRIIb obtained in the subsequent assays can’t be approximated to apparent affinity values and instead should be considered as qualitative indicator of the binding.

### Binding of six FcγRs to human IgG is specific and subclass dependent

**Figure 3:**
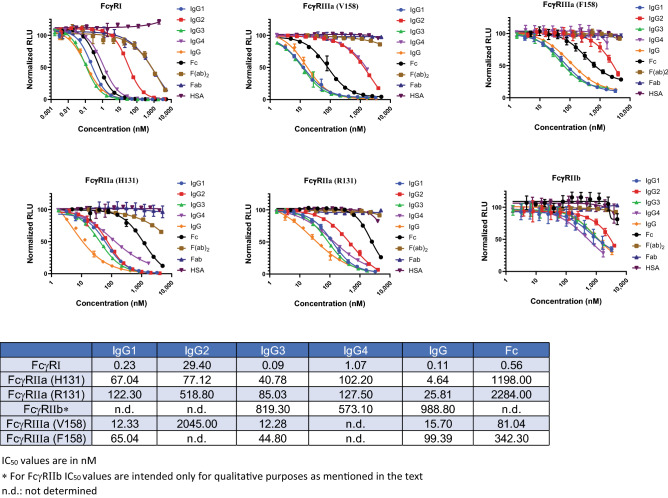
Dose dependent inhibition curves generated with six different FcγR assays. Four different set of samples were tested to show the specificity and subclass specific binding. Analytes tested are **(1)** human IgG subclasses IgG1, IgG2, IgG3, IgG4; **(2)** human IgG; **(3)** Fc, Fab, and F(ab)_2_ domains; and **(4)** human serum albumin (HSA). Data represent the mean ± standard error of triplicate experiments. IC_50_ (nM) values calculated from the inhibition curves are shown in the Table. IC_50_ values are in nM. *For FcγRIIb IC_50_ values are intended only for qualitative purposes as mentioned in the text. *n.d.* not determined.

FcγRs are known to bind various IgG subclasses with different affinities, so, for the first evaluation we tested human IgG1, IgG2, IgG3, IgG4, along with polyclonal IgG for binding to all six FcγRs and calculated IC_50_ values (Fig. 3). There are several key observations from this dataset. We show that FcγRI is the tightest binder whereas FcγRIIb has the weakest affinities for all the IgG subclasses. Relative affinities for binding of various subclasses to FcγRI were IgG3 > IgG1 > IgG4 > > > IgG2. IgG2 is generally considered to be non-binding to FcγRI but in our assay it had a measurable binding, although affinity was two log-order weaker than that of IgG3. For the H131 and R131 variants of FcγRIIa receptors, the relative binding affinities were IgG3 > IgG1 > IgG4≈IgG2. In general, the H131 variant had slightly higher affinities compared to R131. Due to the very weak affinities of IgG for FcγRIIb, IC_50_ values could not be accurately calculated but it was still possible to rank order the various subclasses as follows: IgG4 > IgG3≈IgG1 >> IgG2. Finally, rank order for FcγRIIIa was IgG3≈IgG1 with no, or negligible binding for IgG2 and IgG4. In addition, as expected V158 variant had four–fivefold higher affinity compared to F158 variant.

We also tested just the Fc domain of the IgG and since FcγRs bind to the lower hinge region and CH2 region of IgG, we expected binding of Fc to be similar to that of IgG. However, to our surprise affinity for FcγRIIa receptors was much weaker for Fc compared to full length IgG and for FcγRIIIa receptors binding of Fc was four–fivefold weaker than full length IgG (Fig. 3).

Overall NanoBiT FcγR assays provided expected rank ordering of binding to various IgG subclasses. We further extended the study and tested the specificity of the assays using analytes that are not expected to bind to the receptors. Three analytes tested were Fab and F(ab)_2_ fragments of IgG along with human serum albumin (HSA) (Fig. 3). No binding was seen with any of the receptors except in case of FcγRI where a very weak but measurable binding was seen with Fab and F(ab)_2_ which we think may be due to very small contamination of intact IgG in the sample. Altogether, our observations indicate that NanoBiT FcγR binding immunoassays are specific for binding to Fc domain of the IgG and the binding is sub class specific.

Besides specificity, an important quality attribute of any assay is its reproducibility and therefore we used human polyclonal IgG (described above) as an internal standard during assay development. Over a year, multiple batches of reagents were made, and collated data indicate a very consistent performance with average and standard deviation of IC_50_ (nM) values of 0.13 ± 0.03; 11.35 ± 5.9; 42.62 ± 25.9; 18.41 ± 3.7; 106.9 ± 17.3 for FcγRI, FcγRIIa (H131), FcγRIIa (R131), FcγRIIIa (V158), and FcγRIIIa (F158) respectively. High %CV in the case of FcγRIIa may be attributed to the lack of upper and/or lower asymptote and related variability in curve fitting^24^. In addition to being rapid and reproducible, NanoBiT assays allow calculation of IC_50_ as well as rank ordering of binding of various Abs to all major classes of FcγRs. We would also like to point out that the binding affinities in this assay are not impacted by surface immobilization induced artifacts.

**Figure 4:**
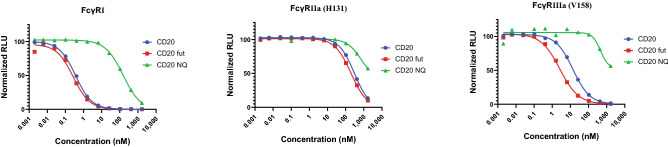
Dose dependent inhibition curves for recombinant anti CD20 IgG1 (CD20), afucosylated anti CD20 (CD20 fut), and deglycosylated anti CD20 (CD20 NQ) on FcγRI, FcγRIIa (H131) and FcγRIIIa (V158). Responses for two variants of FcγRII and FcγRIII were similar therefore only one variant is shown here.

Finally, we tested the assays for their ability to detect changes in Fc/FcγR affinities in response to modification in the Fc domain. It is well known that sugar groups at the hinge region of antibodies in close proximity to FcγR binding site can significantly impact binding therefore we used a commercially available panel of anti CD20 IgG1 recombinant antibodies available in native form, deglycosylated, and afucosylated forms and tested them in FcγR assay (Fig. 4). Deglycosylated IgG1 had a significantly lower affinity for all three receptors compared to native IgG whereas removing the fucose leads to improvement in the affinity by about 1.5-fold and 6.0-fold for FcγRIIa (H131) FcγRIIIa (V158) respectively. Improvement for FcγRI was minimal. These trends are in line with the reported impact of Fc glycans on the FcγRs^25^.

### Binding of therapeutic antibodies to FcγRs

**Figure 5:**
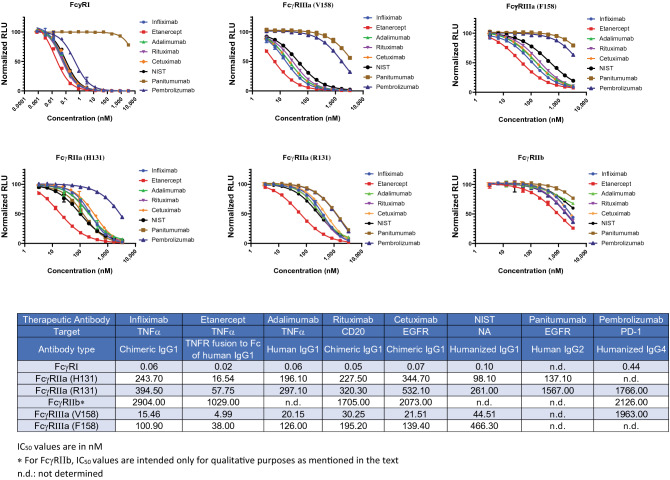
Dose dependent inhibition curves generated with six different NanoBiT FcγR assays. A diverse set of therapeutic antibodies were tested along with one NIST mAb standard. Data represent the mean ± standard error of triplicate experiments. IC_50_ (nM) values calculated from the inhibition curves are shown in the Table. IC_50_ values are in nM. *For FcγRIIb, IC_50_ values are intended only for qualitative purposes as mentioned in the text. *n.d.* not determined.

Data presented above demonstrated specificity and reproducibility of NanoBiT FcγR assays for various receptors and IgG subclasses, and we also show that this solution-based assay allows for rapid and simple determination of binding affinities. To further validate the assays, we tested a set of well characterized therapeutic monoclonal antibodies to see if the affinities correspond to their antibody subclasses. The therapeutic antibody set comprise of three different subclasses (IgG1, IgG2, IgG4), three antibody types (chimeric, humanized, and human), four target types (TNFα, CD20, PD1, EGFR), one Fc fusion protein and one NIST standard. Experiments with these eight antibodies generated 48 IC_50_ values (Fig. 5). Several trends seen in this panel are similar to those seen in previous experiments and include: (a) FcγRI has the highest affinity whereas FcγRIIb has the lowest affinity among six receptors; (b) affinity of FcγRIIa (H131) is only slightly higher compared to R131 variant for IgG1 antibodies; (c) for same IgG1 panel, FcγRIIIa (V158) has six–tenfold higher affinity compared to FcγRIIIa (F158). However, there were some observations distinct to this panel including minimal or no binding of Panitumumab (IgG2) to any of the receptors except for FcγRIIa (H131) and that Pembrolizumab (IgG4) strongly interact only with FcγRI. In addition, among the five therapeutic IgG1 molecules tested in this section, Etanercept had noticeably higher affinity to all the receptors (p < 0.05) when compared to other four IgG1 drugs. This observation is interesting because, although, Etanercept is a Fc fusion of TNF receptor (TNFR) protein, its Fc domain is similar to four IgG molecules, and we were expecting similar affinities for all five molecules.

**Figure 6:**
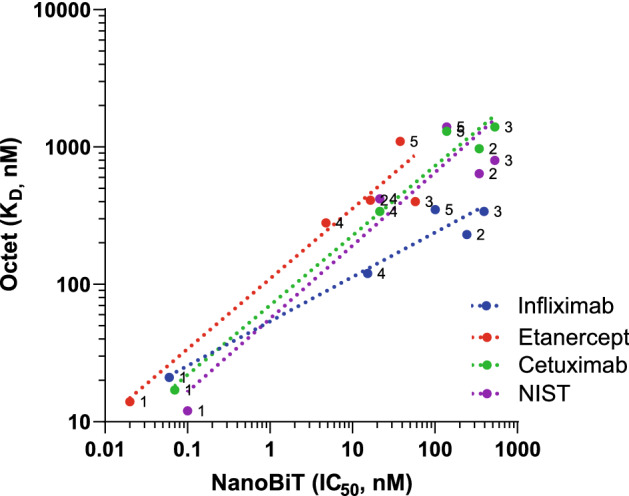
Correlation plot between affinity (IC_50_) determined using NanoBiT assays and affinity (K_D_) determined using BLI platform. IC_50_ (nM) and K_D_ (nM) were determined for binding of Infliximab, Etanercept, Cetuximab and NIST to **(1)** FcgRI, **(2)** FcgRIIa (H131), **(3)** FcgRIIa (R131), **(4)** FcgRIIIa (V158), and **(5)** FcgRIIIa (F158).

We further took a subset of four antibodies—Infliximab, Etanercept, Cetuximab, and NIST—and measured their affinity for FcγRI, FcγRIIa (H131), FcγRIIa (R131), FcγRIIIa (V158), and FcγRIIIa (F158) using BLI, a traditional biosensor platform frequently employed for biomolecular affinity measurements. Affinity measurements were made by immobilizing biotinylated receptor on the Streptavidin sensor and flowing various antibodies over the sensor. K_D_ values were determined from steady state measurement (Table 2). Although the absolute affinity values differ, but the trends on the BLI are similar to NanoBiT FcγR assay with antibodies showing: (a) highest binding affinities of FcγRI; (b) slightly higher affinity with FcγRIIa (H131) compared to FcγRIIa (H131); and (c) three–fourfold higher affinity for FcγRIIIa (V158) compared to FcγRIIIa (F158) (Fig. 6). A discrepancy between the two platform was that Etanercept had higher affinity across all the FcγRs when measured using NanoBiT but not when measured on BLI. It is worth noting that on both the platform, binding affinity for a specific FcγR (except for FcγRI) can vary three–fourfold even though Fc sequence of all IgG1 mAbs have identical sequence close to the hinge and CH2 domain where receptors are known to bind.

**Table 2:** K_D_ (nM) values of four antibodies against five different FcγRs determined using BLI.

Therapeutic antibody	Infliximab	Etanercept	Cetuximab	NIST
Type	TNFα	TNFα	EGFR	NA
Antibody type	Chimeric IgG1	TNFR fusion to Fc of human IgG1	Chimeric IgG1	Humanized IgG1
FcγRI	21	14	17	12
FcγRIIa (H131)	230	410	970	640
FcγRIIa (R131)	340	400	1400	800
FcγRIIIa (V158)	120	280	340	420
FcγRIIIa (F158)	350	1100	1300	1400

### Intravenous IgG (IVIg) and FcγRs

**Figure 7:**
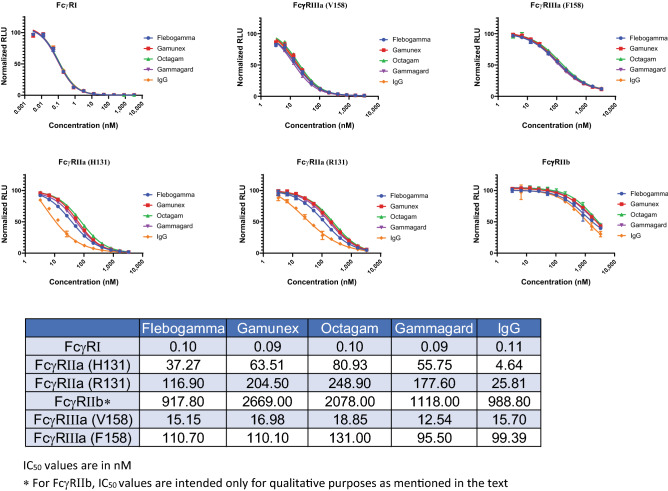
Dose dependent inhibition curves generated with six different NanoBiT FcγR assays. Four commercial IVIg preparation were tested. Inhibition curve for IgG, generated earlier (Fig. 3), is also included for comparison. Data represent the mean ± standard error of triplicate readings. IC_50_ (nM) values calculated from the inhibition curves are shown in the Table. IC_50_ values are in nM. *For FcγRIIb, IC_50_ values are intended only for qualitative purposes as mentioned in the text.

IVIg are widely used therapeutic modalities for treatment of autoimmune diseases and among many postulated mechanism-of-action, one is their interaction with various Fc receptors including FcγRs and FcRn. IVIg are obtained from the plasma of a large pool of thousands of healthy donors to ensure a diverse repertoire, however, IVIg from different manufacturers may differ in their final composition due to production processes. To understand if commercial IVIg preparations will vary in their Fc binding characteristics we measured affinities of four IVIg preparations using NanoBiT FcγR assays (Fig. 7).

In general, the trends seen earlier regarding relative binding affinity of IgG to various FcγRs (FcγRI > FcγRIIIa (V158) > FcγRIIa (H131) > FcγRIIIa (F158) ≈ FcγRIIa (R131) >> FcγRIIb) still holds. In addition, affinities of various IVIg preparations fall within a narrow range for FcγRI and FcγRIIIa but the affinities for FcγRIIa had a wider spread. Interestingly, when we compared the IC_50_ values of IVIg with the research grade pAb used earlier (Fig. 3) FcγRI and FcγRIII (F158 and V158) had similar affinities but affinities for FcγRIIa (H131) and FcγRIIa (R131) were almost 12-fold and sevenfold lower respectively.

## Discussion

Building on our previous work^13^ of developing bioluminescent immunoassay to decipher the interaction between IgG and FcRn, we developed assays to measure the affinity between IgG and FcγRs. The homogeneous format of these assay means that there are no washing steps and therefore, assays are easy and can be performed rapidly in multi-well plates. Most of the data presented in this work was generated using very small sample volume (8.0 µL) and assays were performed manually. But assays can be easily automated to 384 or 1536 well format to further reduce the sample volume and perform a high-throughput screening. As a result, it is possible to gather large data set to get a holistic picture of the Fc/FcγR interaction during the antibody engineering process. Moreover, this is a solution-based assay, eliminating the need for immobilization of molecules and minimize artifacts related to surface properties. Widely divergent Fc/FcγR affinity values, sometimes varying by more than a log order, have been reported when surface immobilization based methods are used^11^. An orthogonal, easy to use solution-based method presented here is not only useful to validate data generated using traditional methods but may also lead to more replicable data.

**Figure 8:**
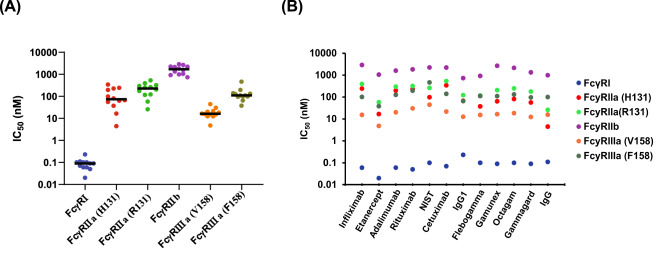
Scatter plot of the IC_50_ values of seven IgG1 antibodies and five different polyclonal IgG plotted two different ways. Lines are the median values. These plots demonstrate the potential advantage of matrix-screening method as a visual aide during large antibody discovery campaigns.

NanoBiT assays reported relative binding of various IgG subclasses to the panel of FcγRs that were similar to those obtained from traditional biosensor platforms and to that expected, based on their functional activity^11,26^. First, relative binding affinities among the FcγRs for human IgG1 was in the order FcγRI > FcγRIIIa (V158) > FcγRIIa (H131) > FcγRIIIa (F158) ≈ FcγRIIa (R131) >> FcγRIIb (Fig. 8A). Second, among subclasses of IgG, binding of IgG3 ≥ IgG1 for all the six receptors, whereas IgG2 and IgG4 had more selective binding. IgG2 only bound with observable or significant affinity to FcγRIIa (H131) and IgG4 bound to FcγRI and two variants of FcγRIIa but did not bind to either variant of the FcγRIIIa. These observations with NanoBiT immunoassays match the data obtained from SPR and Luminex platform^11,14^. Lack of binding of IgG2 and IgG4 to FcγRIIIa means they cannot activate the immune functions like ADCC and are preferred in some therapeutic modalities. The first example is the treatment of metastatic cancer caused by the overexpression of EGFR using two anti EGFR antibodies, Cetuximab (IgG1) and Panitumumab (IgG2). Although both antibodies have the same MOA of blocking the binding of EGF to EGFR, they belong to different subclasses and interact with FcγRs in disparate manner^27^. Cetuximab being an IgG1 can activate ADCC through recruitment of natural killer (NK) cells. The MOA of Panitumumab, however, is assumed to be independent of Fc receptor interactions and primarily involves Panitumumab acting as an antagonist and preventing binding of EGF to EGF receptor (EGFR)—inhibiting dimerization of EGFR and downstream signaling which ultimately results in reduced cell proliferation and increased apoptosis^28^. However, our data indicate Panitumumab does binds to FcγRIIa (H131) and so what effect if any, this interaction has on the drug efficacy is not known. Both these drugs have similar clinical efficacy, but metastatic cancer respond differently to these two drugs possibly due to their distinct MOAs. Correlating comprehensive binding and structural data with clinical data may offer new ways of targeting diseases and preventing resistance to drugs. The second example, where reduced binding to FcγRIIIa, in this case of IgG4, has been an advantage, is in the case of immunotherapy. MOA of the immunotherapeutic drugs, also called checkpoint inhibitors, is to block PD-1, PD-L1, or CTLA-4 proteins on the immune cells and increase cytotoxic T-cell activity against cancer cells. For these drugs any activation of ADCC through FcγRIIIa interaction will be detrimental, as a result these drugs (e.g. Pembrolizumab, Nivolumab, etc.) belong to the IgG4 subclass.

NanoBiT FcγR assays can correctly capture the changes in binding affinity to various FcγRs in response to deglycosylation and afucosylation. This is an important feature, as glycoengineering of therapeutic antibody candidates impacts the effector functions^29,30^ and several Fc modified antibodies have been approved for therapeutic use and many more are in clinical trials^31^. In our study, afucosylation increased the affinity of IgG to FcγRIIa, and FcγRIIIa by 1.5-fold and 5.0-fold respectively and deglycosylation significantly reduced the binding but did not eliminate it. The fact that N297Q variant in absence of any glycosylation still retained significant binding especially to FcγRIIa is interesting and against the reported results where this mutation abolished the binding to all the FcγRs^32^. We don’t know the exact reason for this discrepancy but speculate that factors other than glycosylation may be driving the interaction. In fact, many antibody variants have been described in the literature^32,33^ where even the glycosylated antibodies have been shown to have reduced or no binding to FcγRs after engineering mutations in CH2 domains. In one such study^33^, a large number of engineered antibodies were evaluated for binding to FcγRs including one CD20 antibodies containing N297Q mutation, similar to the one used by us. Study showed a measurable although very low binding to FcγRs including FcγRIIa highlighting that our observation is not completely anomalous. In another study with bacterial IgG display system, an aglycosylated antibody was isolated which instead of having a low or negligible binding to FcγR instead had 160-fold improved affinity for FcγRIIa (R131) due to mutations in Fc domain. Such observations highlight the fact that each FcγR interacts with Fc domain in a unique but slightly different way and may be responsible for our observations. However, additional studies are needed to validate our observations. The assays as presented here, which enable the screening of libraries of antibodies against multiple receptors, when combined with orthogonal biochemical and cell based functional assays can identify novel FcγR interactions and identify promising therapeutic candidates.

We compared the NanoBiT platform with the BLI using a small subset of four antibodies of IgG1 subclass. Our approach for the BLI was to use biotinylated FcγRs on streptavidin chip and then flowing antibodies over it as this format has been reported to minimize artifacts^34^. The affinity of IgG1 for various FcγRs obtained on BLI were within the reported range and, more importantly, affinity ranking was as expected^11^ and correlated well with the IC_50_ values obtained using NanoBiT. A notable difference between two assays was in the case of Etanercept in which the NanoBiT platform showed a significantly higher affinity of Etanercept compared to other three antibodies, whereas on BLI there was no trend among four molecules. Observation on NanoBiT platform is unexpected since all four antibodies share identical Fc domain^13^ where FcγRs binds. In fact, excluding Etanercept, rest of the IgG antibodies do have affinities within a narrow range. The key difference between Etanercept and the rest of the IgG1 antibodies is that Etanercept is a Fc fusion of TNFR whereas other three are traditional IgG. Thus, it could be argued that antigen binding domain may be impacting the affinity measurement either on NanoBiT or in BLI and may also be responsible for anomalous data seen with Fc fragment of IgG (see additional discussion below). In fact, conflicting data for Etanercept have been reported when measuring affinity with FcRn using traditional biosensing platform and could be attributed to the biosensor assay format. Clinically, efficacy of therapeutic TNF-inhibitors (e.g., Etanercept, Infliximab and Adalimumab) varies depending on the type of inflammatory disease (e.g., Crohn, Rheumatoid arthritis etc.) and MOA and role of Fc effector function is not well defined^35^. Therefore, it is not possible to pinpoint whether NanoBiT or BLI data are correct, but it does reinforce the need for orthogonal methods for validating binding data especially for structurally different molecules as in the case for Etanercept.

Another unexpected observation was the significantly weak binding of the human IgG Fc fragment compared to full length IgG for FcγRs (Fig. 3). Since FcγRs binds at the lower hinge region and the CH2 domain of the Fc fragment, we were expecting the affinities of Fc domain and full-length IgG to be similar. In fact, with our earlier study on NanoBiT Fc/FcRn binding, Fc domain and IgG did have similar binding to FcRn but in that case FcRn binds further down from hinge region at CH2-CH3 domain of Fc. The difference in the affinities of Fc fragment and full-length IgG obtained with NanoBiT FcγR raises an intriguing possibility of Fab fragment influencing the Fc/FcγR binding, a topic not well investigated for FcγR. In fact, a recent report studied interaction of IgG with FcγRIIIa using BLI, isothermal calorimetry, hydrogen/deuterium exchange mass spectrometry, and crosslinking mass spectrometry and concluded that Fab fragment can stabilize the IgG/FcγRIIIa interaction and impacts the affinities. They compared the binding affinities of full-length IgG and Fc fragment for FcγRIIIa and found Fc to have a lower binding affinity, similar to our observation. Influence of Fab domain on interaction with Fc receptors has been extensively debated in the case of FcRn with studies indicating significant role of Fab fragment in the binding^36,37^, whereas other, including our own, shows no impact of Fab fragment^13,38^. Another possible reason could be differences in glycosylation pattern which are known to impact Fc/FcγR binding as shown in this study as well. Additional systematic studies using a panel of native and deglycosylated IgG (mAb and pAb) and their Fc and F(ab)_2_ fragments prepared from the same IgG is needed to decipher the impact of F(ab)_2_ domain and glycosylation on Fc/FcγR binding.

Unlike therapeutic antibodies that are designed to trigger or abolish certain Fc effector functions, IVIg are complex mixtures and may impact multiple Fc receptors simultaneously. We compared four different commercially available IVIg products for their binding to FcγRs and found them to be very similar. This is not surprising since IVIg is prepared from plasma pooled from thousands of healthy individual and any differences in the binding profile of an individual IgG sample will be lost. It was however interesting to observe higher affinity of a research grade IgG for FcγRIIa compared to IVIg (p < 0.05). We don’t know if the purification process or some unique feature of serum from which IgG was purified is responsible for the higher affinity, but this does point to the possibility that polyclonal antibody binding profile to specific FcγRs may differ and therefore profiling antibodies against all the Fc receptors can be of value in understanding the efficacy of antibody therapies. Recent developments and especially work from Alter et al.^39–41^ on systems serology has highlighted the importance of such screening in better understanding of infectious diseases and on the efficacy of vaccines. In this regard a simple assay as described here can be an enabling tool.

An advantage of the parallel screening approach used in this work is the ability to quickly identify unique attributes of the antibodies. For example, as seen in Fig. 8A, antibody affinities to FcγRIIa (H131) vary by more than 75-fold whereas variation is around eightfold for FcγRIIIa (V158) for the same set. Despite the fact that sequence of IgG1 antibodies in the antibody hinge and CH2 domain are similar such wide variation in affinities can offer insights enabling better antibody therapies. For example, elimination of IgG/FcγR receptor interaction is a desirable characteristic to prevent unwanted inflammatory response for antibody therapies, and mutations in Fc domain as well as glycoengineering are some of the approaches used to achieve this goal. But often these modifications can reduce and not eliminate the binding to the receptors or may eliminate binding to a specific receptor while leaving other interactions unchanged. Our study highlighted that antibodies with identical Fc sequence interact differently with various Fc receptor and points to other factors such as Fab may also play a role. Such insights may allow a tailored approach to antibody engineering to achieve a specific goal. We do want to point out that observations made in our studies need to be validated using cell based and in-vivo studies. Similarly scatter plot shown in Fig. 8B provide a ‘visual picture’ of the set of IgG1 and polyclonal antibodies and it is easy to identify Etanercept and the research grade polyclonal IgG as a possible outlier worth further investigation. A similar approach when implemented during antibody drug discovery or antibody engineering process can quickly narrow down the promising candidates for secondary screening in more relevant models like cell-based assays. A desirable feature, not explored in this paper, that will further enhance the throughput of the parallel screening approach is to find an alternative to generating full inhibition curves to extract IC_50_ values.

NanoBiT FcγR assays like other biochemical assays have limitations. First, Fc receptors are membrane bound proteins but in biochemical assays a soluble fragment of the receptor is used and therefore will miss any role of cell membrane in the binding Fc/FcγR binding. Second, our assays measure the binding of monomeric IgG to FcγRs where as in-vivo FcγRIIa and FcγRIIIa only bind antibodies in the form of immune complexes. However, biochemical assays can sensitively capture changes in Fc/FcγR binding resulting from changes in glycan structure, amino acid modification in the Fc domain or small conformation changes in IgG^42–44^ and such changes often correlates well with in-vivo data. Therefore, regulatory agencies like FDA/EUA require submission of Fc/FcγR biochemical binding data especially in cases of biosimilar approvals.

In conclusion, NanoBiT FcγR assays when combined with the FcRn assay described in our earlier work, provide easy-to-use, specific, and reproducible method to screen antibodies for a wide variety of critical quality attributes influenced by antibody Fc domain and will benefit research into next generation vaccines and antibody drugs.

## Data Availability

The datasets generated during and/or analyzed during the current study are available from the corresponding authors on reasonable request.

## Abbreviations

FcγRs: Fc-gamma receptors
HSA: Human serum albumin
SPR: Surface plasmon resonance
BLI: Biolayer interferometry
IgG: Immunoglobulin
Fc domain: Fragment crystallizable domain
F(ab)_2_: Fragment antigen binding domain
TNFR: TNF Receptor
MOA: Mechanism of action
LgBiT: 18KDa Large BiT polypeptide
SmBiT: 11-Aa Small BiT peptide
Tracer: IgG-LgBiT
Target: FcγR-SmBiT
mAb: Monoclonal antibody
pAb: Polyclonal antibody
IVIg: Intravenous immunoglobulin
ADCC: Antibody dependent cellular cytotoxicity
ADCP: Antibody dependent cellular phagocytosis
Fc/FcγR: Interaction of IgG as well as Fc fusion proteins with FcγRs

## References

[1] Kang TH, Jung ST (2019). Boosting therapeutic potency of antibodies by taming Fc domain functions. Exp. Mol. Med..

[2] Nagelkerke SQ, Kuijpers TW (2014). Immunomodulation by IVIg and the role of Fc-Gamma receptors: Classic mechanisms of action after all?. Front. Immunol..

[3] Prevot J, Jolles S (2020). Global immunoglobulin supply: steaming towards the iceberg?. Curr. Opin. Allergy Clin. Immunol..

[4] Stephenson KE (2021). Immunogenicity of the Ad26.COV2.S vaccine for COVID-19. JAMA.

[5] McMahan K (2021). Correlates of protection against SARS-CoV-2 in rhesus macaques. Nature.

[6] Selva KJ (2021). Systems serology detects functionally distinct coronavirus antibody features in children and elderly. Nat. Commun..

[7] Daeron M (1997). Fc receptor biology. Annu. Rev. Immunol..

[8] Nimmerjahn F, Ravetch JV (2008). Fcgamma receptors as regulators of immune responses. Nat. Rev. Immunol..

[9] Nagelkerke SQ, Schmidt DE, de Haas M, Kuijpers TW (2019). Genetic variation in low-to-medium-affinity Fcgamma Receptors: Functional consequences, disease associations, and opportunities for personalized medicine. Front. Immunol..

[10] Pyzik M, Rath T, Lencer WI, Baker K, Blumberg RS (2015). FcRn: The architect behind the immune and nonimmune functions of IgG and albumin. J. Immunol..

[11] Forest-Nault C, Gaudreault J, Henry O, Durocher Y, De Crescenzo G (2021). On the use of surface plasmon resonance biosensing to understand IgG-FcgammaR interactions. Int. J. Mol. Sci..

[12] Drake AW (2012). Biacore surface matrix effects on the binding kinetics and affinity of an antigen/antibody complex. Anal. Biochem..

[13] Nath N, Godat B, Flemming R, Urh M (2021). Deciphering the interaction between neonatal Fc receptor and antibodies using a homogeneous bioluminescent immunoassay. J. Immunol..

[14] Boesch AW (2014). Highly parallel characterization of IgG Fc binding interactions. MAbs.

[15] Brown EP (2017). Multiplexed Fc array for evaluation of antigen-specific antibody effector profiles. J. Immunol. Methods.

[16] Seo N (2018). Analytical and functional similarity of Amgen biosimilar ABP 215 to bevacizumab. MAbs.

[17] Handlogten MW (2020). Prevention of Fab-arm exchange and antibody reduction via stabilization of the IgG4 hinge region. MAbs.

[18] Basgalupp S (2021). Diagnostic properties of three SARS-CoV-2 antibody tests. Diagnostics.

[19] Janaka SK (2021). Predicting the efficacy of COVID-19 convalescent plasma donor units with the Lumit Dx anti-receptor binding domain assay. PLoS ONE.

[20] Li B (2021). High-Throughput NanoBiT-based screening for inhibitors of HIV-1 Vpu and Host BST-2 protein interaction. Int. J. Mol. Sci..

[21] Alves J (2021). A bioluminescent and homogeneous SARS-CoV-2 spike RBD and hACE2 interaction assay for antiviral screening and monitoring patient neutralizing antibody levels. Sci. Rep..

[22] Chau CH, Steeg PS, Figg WD (2019). Antibody-drug conjugates for cancer. Lancet.

[23] Cheng Y, Prusoff WH (1973). Relationship between the inhibition constant (K1) and the concentration of inhibitor which causes 50 per cent inhibition (I50) of an enzymatic reaction. Biochem. Pharmacol..

[24] Sebaugh JL (2011). Guidelines for accurate EC50/IC50 estimation. Pharm. Stat..

[25] Li T (2017). Modulating IgG effector function by Fc glycan engineering. Proc. Natl. Acad. Sci. USA.

[26] Bruhns P (2009). Specificity and affinity of human Fcgamma receptors and their polymorphic variants for human IgG subclasses. Blood.

[27] Garcia-Foncillas J (2019). Distinguishing features of cetuximab and panitumumab in colorectal cancer and other solid tumors. Front. Oncol..

[28] Dubois EA, Cohen AF (2009). Panitumumab. Br. J. Clin. Pharmacol..

[29] Trastoy B (2022). Sculpting therapeutic monoclonal antibody N-glycans using endoglycosidases. Curr. Opin. Struct. Biol..

[30] Li W, Zhu Z, Chen W, Feng Y, Dimitrov DS (2017). Crystallizable fragment glycoengineering for therapeutic antibodies development. Front. Immunol..

[31] Liu R, Oldham RJ, Teal E, Beers SA, Cragg MS (2020). Fc-engineering for modulated effector functions-improving antibodies for cancer treatment. Antibodies.

[32] Sazinsky SL (2008). Aglycosylated immunoglobulin G_1_ variants productively engage activating Fc receptors. Proc. Natl. Acad. Sci. USA.

[33] Wilkinson I (2021). Fc-engineered antibodies with immune effector functions completely abolished. PLoS ONE.

[34] Abdiche YN (2015). The neonatal Fc receptor (FcRn) binds independently to both sites of the IgG homodimer with identical affinity. MAbs.

[35] McRae BL (2016). Fc receptor-mediated effector function contributes to the therapeutic response of anti-TNF monoclonal antibodies in a mouse model of inflammatory bowel disease. J. Crohns Colitis.

[36] Schoch A (2015). Charge-mediated influence of the antibody variable domain on FcRn-dependent pharmacokinetics. Proc. Natl. Acad. Sci. USA.

[37] Jensen PF (2017). A two-pronged binding mechanism of IgG to the neonatal Fc receptor controls complex stability and IgG serum half-life. Mol. Cell Proteom..

[38] Foss S (2016). Enhanced FcRn-dependent transepithelial delivery of IgG by Fc-engineering and polymerization. J. Control Release.

[39] Francica JR (2021). Protective antibodies elicited by SARS-CoV-2 spike protein vaccination are boosted in the lung after challenge in nonhuman primates. Sci. Transl. Med..

[40] Chung AW, Alter G (2017). Systems serology: Profiling vaccine induced humoral immunity against HIV. Retrovirology.

[41] Ackerman ME, Barouch DH, Alter G (2017). Systems serology for evaluation of HIV vaccine trials. Immunol. Rev..

[42] Sondermann P, Kaiser J, Jacob U (2001). Molecular basis for immune complex recognition: A comparison of Fc-receptor structures. J. Mol. Biol..

[43] Lu J (2015). Structure of FcgammaRI in complex with Fc reveals the importance of glycan recognition for high-affinity IgG binding. Proc. Natl. Acad. Sci. USA.

[44] Siberil S (2006). Molecular aspects of human FcgammaR interactions with IgG: Functional and therapeutic consequences. Immunol. Lett..

